# Evaluation of Osseointegration and Bone Healing Using Pure-Phase β - TCP Ceramic Implant in Bone Critical Defects. A Systematic Review

**DOI:** 10.3389/fvets.2022.859920

**Published:** 2022-07-12

**Authors:** Daniel Cardoso Garcia, Larissa Eckmann Mingrone, Marcelo Jorge Cavalcanti de Sá

**Affiliations:** ^1^Doctoral Student at the Department of Small Animal Surgery, Faculty of Veterinary Medicine, Federal University of Campina Grande (UFCG), Patos, Brazil; ^2^Small Animal Surgery Service, Animal Care Barueri Clínica Veterinária, São Paulo, Brazil; ^3^Department of Small Animal Surgery, Faculty of Veterinary Medicine, Federal University of Campina Grande (UFCG), Patos, Brazil

**Keywords:** biomaterials, β - TCP, osseoconduction, osseointegration, bone healing, synthetic implant

## Abstract

**Background:**

The gold standard for osseointegration remains the autogenous bone graft, but biomaterials such as Beta - tricalcium phosphate (β - TCP) in its pure-phase showed promising results to be practical bone substitutes. This kind of implants are optimal candidates for bone integration due to their osseoconductive, biocompatibility, bioactivity, and absorptive properties.

**Methods:**

A systematic review was conducted using 5 databases (Cochrane Library, PubMed, Scielo, Medline-Bireme and Google Scholar) for searching published studies between January 1st 2011 and June 15th 2021. Only clinical and experimental studies, and case reports were included in this research. Human and animal studies published only in Portuguese or English with clinical, radiologic, and histologic evidence of new bone formation, osseoconduction, and osseointegration were included. This systematic review was reported according to PRISMA guidelines.

**Results:**

Approximately 14.554 articles were initially found, but after advanced searching using specific including and excluding keywords, matching Boolean operators “AND,” “OR” and “NOT,” and after excluding duplicates, a total of 12 articles were included for this systematic review, including experimental works, a retrospective study, a randomized controlled clinical study, a randomized prospective study, a prospective observational study, and a case report. All articles showed 100% effectiveness in bone integration after β - TCP implantation by clinical, image and/or histologic assessment. Implant shape and porosity seem to have influence in osseointegration process. β - TCP can give predictable, sustainable, and adequate new bone formation with the least infection rates in implant placement cases and patient morbidity, which is the current goals for bone integration, augmentation and replacement.

**Conclusion:**

β - TCP in its pure-phase is widely used in dentistry and maxillofacial surgery, but there is a lack of information about the use of this biomaterial for filling critical segmental defects of long bones in veterinary medicine. In this area, only experimental studies in small defects were carried out, with no clinical cases performed in animals with a longer observation time. β - TCP can produce predictable, sustainable, and adequate bone formation, with minimal infection rates and low patient morbidity. But more clinical studies in the future, demonstrating specific metric measurements in relation to bone consolidation, as well as showing results using other shapes of this implant are needed to evaluate further in depth osseoconductive and osseointegrative characteristics of this biomaterial, in order to develop new comparisons and quantitative analyses for its use in veterinary medicine as a bone replacement.

## Introduction

Restoring lost tissue through the application of tissue engineering principles is a complex process that involves interaction between cells, growth factors, and scaffolds (1). What still remains a challenge is how to improve the integration of the newly formed bone with the surrounding tissues (2).

Replacement of part of the bone tissue is often necessary in the surgical routine, whether due to fractures, bone neoplasms, or orthopedic diseases that lead to bone loss (3). For over 100 years, the gold standard and the most favorable results for bone repair has been the transplantation of autologous bone from the iliac crest, providing the advantages of osseoconductive, osseoinductive, and osteogenic actions at the implantation site (4–6). It has been reported that the release of growth factors such as platelet-rich plasma (PRP) and transforming growth factor-β (TGF-β) associated with early vascularization of the donor bone allows for remodeling within 4–6 months after placement of the implant (5). However, this procedure can be hampered by donor site morbidity (7), limited graft availability (4, 5), donor site infection (4, 8), need for additional surgical intervention (8, 9), excessive resorption (4, 8), and greater stress for both the surgeon and the patient (5).

As readily available and low-cost alternatives, allografts, xenografts, and alloplastics have been used (9, 10). Of all these options described, all have been shown to have osseoconductive properties (except lyophilized demineralized bone allograft, which is an osseoinductive material). However, allografts carry the risk of problems integrating with the surrounding bone tissue and, more importantly, they can induce immune responses in the recipient with unpredictable consequences (4). Its preparation requires sterilization and deactivation of proteins normally found in healthy bone, which eliminates the extracellular matrix (7), as well as bone growth factors, proteins, and other bioactive materials necessary for osseoinduction (7). They have some disadvantages, including the demineralization process and the need to use frozen tissue. The use of dehydrated human bone is also controversial. Clinically and histologically, allografts present very good results (7, 11). On the other hand, such materials present the risk of transmitting infectious diseases. This fact led to greater interest in the research and development of synthetic biomaterials similar to bones (9).

Natural and synthetic bone substitutes are widely used for bone regeneration due to its biocompatibility, osseoinductive and osseoconductive effects, and absence of risk of antigenicity. These materials concurrently serve as a “scaffold” and as osteogenesis stimulators, facilitating bone regeneration, and are often replaced by newly formed bone tissue after graft or implant resorption (8, 9, 11). These biomaterials are provided mechanical, and inductive support transformers to tissues in reflex, features related to bone substitutes (9).

In this sense, the biomaterial is very capable of promoting bone replacement in order to avoid the use of grafts or bone transplants (3). Synthetic products were formed as replacements for bioinert compounds, several used over decades with the primary purpose of filling and augmenting bone in skeletal facilities. It is known that phosphate compounds have affinity for specific tissues such bones and showed promising results among the various alloplastic materials used for grafting (6).

Alloplastics do not have osteogenic or osseoinductive properties but are osseoconductive materials (7). On the other hand, these materials are widely available, inexpensive, and do not have the potential to transmit disease or infection (7, 10). Furthermore, as they are synthetic, their chemical composition and fabrication can be increasingly manipulated to mimic the characteristics of natural bone (10).

Ideally, as a bioactive calcium phosphate (CaP) ceramic for use in bone material, it exhibits a good behavior in relation to bone bonding, stimulating the formation of bone tissue at the interface between the biomaterial and the features, and to add these features, also must show a high rate of degradation, thus finding a balance between rapid bone formation and rapid biodegradation (8).

CaP bioceramics in the form of granules or microporous blocks stand out in the researches as biomaterials for defect repair and bone tissue reconstruction, as mentioned by different authors, who observed bone neoformation when these biomaterials were applied *in vivo* (12).

Microporous CaP bioceramics are manufactured with architecture similar to structure of bone tissue, formed by fine grains, with interconnected microporosity, which helps in the mechanism of cell adhesion and proliferation through osseoconduction, osseointegration, and formation of new bone (12, 13).

It is verified that the performance of these bioceramics in the process of bone neoformation is associated with the characteristics of bioactivity, solubility, wettability, interconnected microporous microstructures, higher superficial areas of grains, and micropores.

These characteristics provide conditions for the adhesion of osteoblasts to the surface of the granulated biomaterial, as well as in the interconnected microporosity throughout the granulated biomaterial (3), leading to biomineralization of bone tissue.

Due to their ability to bond to bone and stimulate bone tissue formation, bioactive CaP ceramics are seen as excellent materials for implantation for candidates who need bone augmentation, filling or replacement (8). Among the biodegradables and osseoconductors biomaterials currently in use and mentioned above, highlights the β - TCP, which is the most popular (2, 8). The β - TCP is reabsorbed completely and has no intrinsic osteogenic or osseoinductive properties (7). However, β - TCP has excellent osseoconductivity and biocompatibility. About its microporosity, pores of 50 μm are sufficient to allow osseoconduction (2). Furthermore, it was reported that the β - TCP surface architecture can stimulate monocyte/macrophage differentiation invaders in osteoclasts, and these cells may be essential for ectopic bone formation (2).

Synthetic bone substitutes, such as β - TCP, are clinically very beneficial because they can avoid surgery on a donor site. Thus, β - TCP is considered an ideal grafting material (5). It has the property of being absorbed by the body while being replaced by bone; in addition, its usefulness has been well-reported in maxillofacial surgery (5) and it is mainly used in dentistry for filling dental sockets and augmenting of the maxillary sinus floor (10). However, the predictability of the material and the change in absorption of the transplanted material remain largely unknown (10). Significant resorption and integration of β - TCP particles are expected 3–6 months after placement (2, 4, 6, 13) allowing for a re-arrangement of trabecular bone during this period (6), but little change is observed after 1.5 years (5). Most β - TCP is biodegraded both by osteoclastic activity after particle breakdown, as well as by chemical dissolution of the molecule into calcium and phosphate components followed by replacement with healthy bone (4, 7).

Thus, an important characteristic of synthetic bone substitutes is their bioactivity. This means that the implants suffer resorption at the same time that occurs new bone formation, resulting in true osseointegration and “restitutio ad integrum” (5, 13). In this context, it is important to state that the so-called “resorbable” bone substitutes, including calcium phosphates, were somewhat unsuccessful in this regard, since they could still be detected years after of their implantation due to inadequate resorption (5). In contrast, some studies demonstrated an accelerated resorption of several types of biomaterials, which was not compensated by the increased formation of new bone at the site. Thus, the choice of the applied biomaterial needs to be made according to the patient's comorbidities, in order to provide the best type of treatment combined with excellent results (8).

## Rationale and Objective

The objective of this article was to carry out a systematic review to evaluate osseointegration and bone consolidation when using the β - TCP ceramic implant in its pure- phase in critical bone defects, concomitantly evaluating other pertinent attributes related to the biomaterial studied. The idea is that we would find literature that would serve as a support and basis for the use of this biomaterial in critical bone defects in small animals, especially in more severe cases with great bone loss, where there will be the need to fill or replace the bone gap created after the loss of bone tissue. We found necessary this kind of study because our group is trying to find a biomaterial such as β - TCP with different shapes and porosities that could be used to fill great fracture gaps in dogs and cats using a synthetic material that is easy to produce and affordable to owners with low budget to treat their pets.

## Materials and Methods

This systematic review adopted and followed the protocols specified by the “Preferred Reporting Items for Systematic Reviews and Meta Analysis” (PRISMA) (14), with the following question elaborated according to the FINGER criteria (15): Is there osseointegration and bone consolidation of long bones critical segmental defects using β - TCP ceramic implant in its pure phase to fill the created defect?

### Criteria for Inclusion and Exclusion of Articles

Using the PICO strategy (P = population/patients; I = intervention/exposure; C = comparison/control; O = “outcome”/outcome/result) (16), articles that met the following criteria were included in this review systematic.

**Population:** Patients (humans or animals) in which the ceramic implant of β - TCP was used in bone defects.

**Intervention:** Use of pure β - TCP ceramic phase in critical bone defects in long bones.

**Comparison:** Other types of grafts and other types of implants for filling, or even defects without filling.

**Outcome:** Osseoconduction, osseointegration, and bone healing of critical bone defects.

### Article Selection Criteria

- Only full articles between January 2011 and June 2021.
- All human and animal studies published in English or Portuguese with clinical, radiological and/or histological evidence of bone neoformation.
- Experimental work, clinical trials, and case reports.

### Exclusion Criteria for Articles

- Abstracts without the presence of full articles.
- Review articles.
- Articles published prior to 2011.
- Articles in languages other than Portuguese or English.

### Study Search and Selection Strategies

A systematic search was carried out exploring the Cochrane Library, PubMed, Scielo, Medline-Bireme and Google Scholar databases for articles that addressed the role of pure-phase β - TCP in osseointegration and bone healing between January 1st 2011 and June 15th 2021. Keywords associated with Boolean operators were used for such searches. For this, the words “β - TCP” OR “Beta TCP” OR “Beta Tricalcium Phosphate” OR “Beta Phosphate Tricalcium” AND “Osseointegration” OR “Osteointegration” were included. Due to the high number of articles in the Google Scholar database, we restricted the search by adding one more keyword filter with the Boolean operator AND and the word “pure-phase,” in addition to using the Boolean operator NOT to the keywords “composite” OR “biphasic” in order to exclude these last two words. For the other databases mentioned above, advanced search was not an issue as there were few articles. The search query was modified for each database if necessary to reach the most relevant studies. Then, data were collected based on the relevance to the study topic and the main objective, through a tool for evaluating titles and abstracts called Rayyan (17), which was performed by 2 independent researchers in a blind way. From this, any conflicts between authors were resolved by reading the full text to determine whether a particular article was selected for inclusion or exclusion in the systematic review.

### Data Extraction

After a first phase of identification and a second phase of screening including an advanced searching of the five databases, one hundred and fourteen studies were chosen as eligible articles. Then the references of these studies were manually searched and checked on Google Scholar. After careful full text reading of the 114 articles, we added only the relevant articles describing about osseointegration using β - TCP in its pure-phase showing image, clinical and/or histologic information. Duplicate articles were excluded. At this point, twelve studies were chosen for data extraction. From the selected articles, relevant information about study design, study population, methodology and results were extracted. Other relevant characteristics related to the implant used in the study were also mentioned. The inclusion of articles in this systematic review was made by mutual agreement between 2 authors.

### Risk of Bias

The methodological quality for the selection of studies was evaluated by two reviewers (DCG and LEM) using the JBI critical appraisal tool (18) for each study individually. The two authors resolved any disagreement regarding the risk of bias of each study by discussion and consensus among reviewers, and if any doubts or disagreements persisted between the first two reviewers, a third reviewer (MJCS) was consulted.

## Results

**Figure 1:**
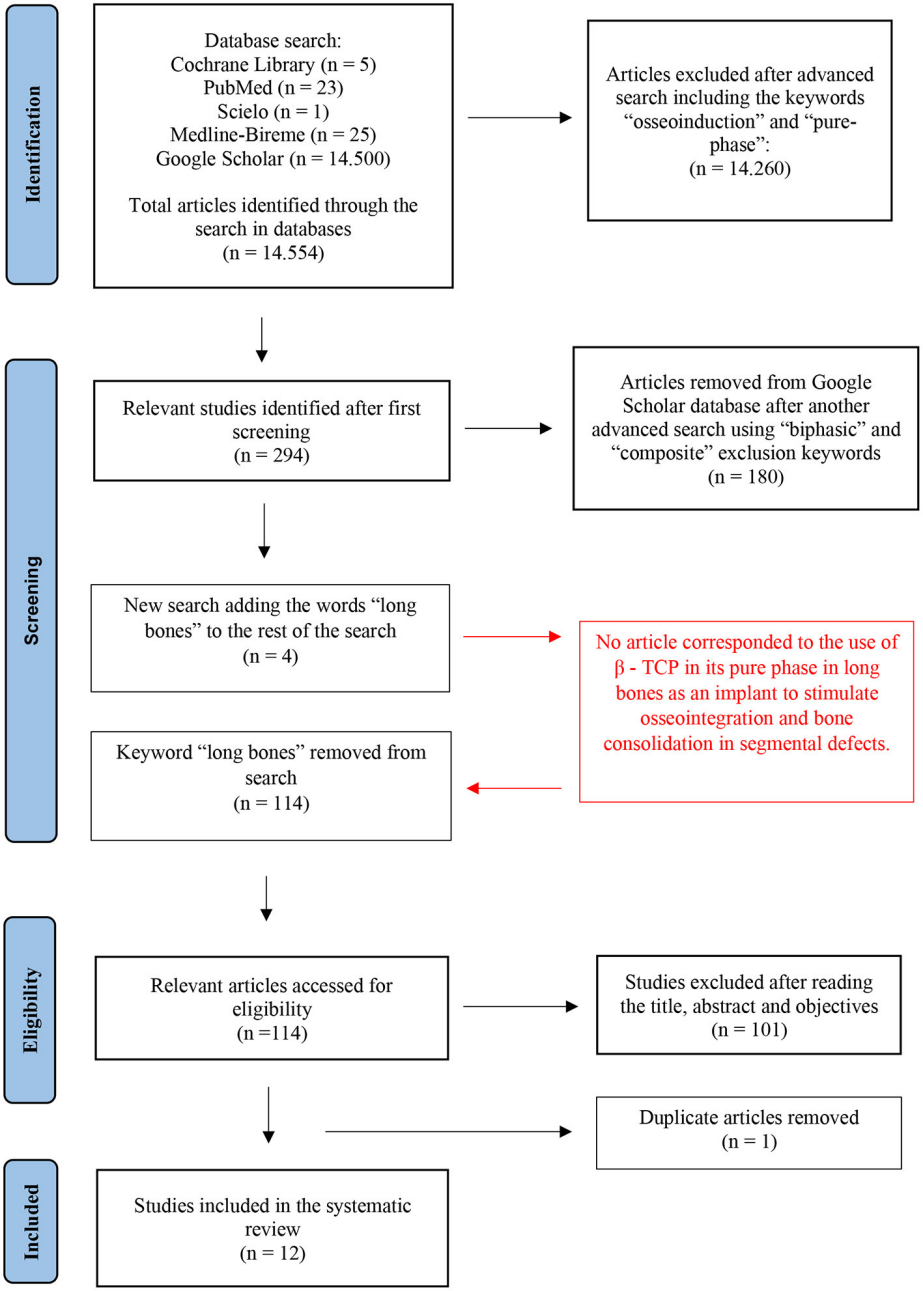
PRISMA flowchart of the search strategy for the data collected for inclusion and exclusion of articles in the systematic review.

A total of 114 articles published between 2011 and 2021 were identified using the databases through a systematic electronic search. After complete text reading and evaluation by two independent reviewers and excluding 1 duplicated article, the full texts of 12 articles were obtained (Figure 1). Below we can identify the number of articles found in each of them:

- Cochrane Library: A total of 5 articles were found by searching the keywords “β - TCP” OR “Beta TCP” OR “Beta Tricalcium Phosphate” OR “Beta Phosphate Tricalcium” AND “Osseointegration” OR “Osteointegration.”
- Pubmed: 23 articles were found using the keywords “β - TCP” OR “Beta TCP” OR “Beta Tricalcium Phosphate” OR “Beta Phosphate Tricalcium” AND “Osseointegration” OR “Osteointegration” in the search.
-Scielo: Only 1 article was found using the keywords “β - TCP” OR “Beta TCP” OR “Beta Tricalcium Phosphate” OR “Beta Phosphate Tricalcium” AND “Osseointegration” OR “Osteointegration.”
- Medline-Bireme: 25 articles were found using the keywords “β - TCP” OR “Beta TCP” OR “Beta Tricalcium Phosphate” OR “Beta Phosphate Tricalcium” AND “Osseointegration” OR “Osteointegration.”
- Google Scholar: Approximately 14.500 articles were found using the keywords “β - TCP” OR “Beta TCP” OR “Beta Tricalcium Phosphate” OR “Beta Phosphate Tricalcium” AND “Osseointegration” OR “Osteointegration” in the searches.

For Google Scholar, refining of searching was needed through the advanced search, in which the Boolean operator AND and the words “osseoinduction” and “pure-phase” were added, 294 articles were found. Then, a new advanced search using the Boolean operator NOT to exclude the terms “composite” OR “biphasic” brought us to a total of 114 articles, excluding other 180 articles. Further refining in article searching, the words “long bones” were included by the Boolean operator OR, associating it with the previously mentioned terms “β - TCP” OR “Beta TCP” OR “Beta Tricalcium Phosphate” OR “Beta Phosphate Tricalcium” AND “Osseointegration” OR “Osteointegration” AND “pure-phase” NOT “biphasic” OR “composite,” and a total of 4 studies were filtered. However, of these four studies, none of the articles corresponded to the use of β - TCP in its pure-phase in long bones as an implant that stimulated osseointegration and bone consolidation in segmental defects, therefore, the words “long bones” were excluded from the search, and after reading the titles and abstracts of the final articles, a total of 12 full articles were included for this systematic review.

The authors of the articles related to each study design, the description of the observation time and the number of patients or animals included in the studies are summarized in Table 1.

**Table 1:** Articles included in the systematic review showing authors, type of study, observation time, and number of patients or animals used in the study.

	**References**	**Study design**	**Time of study**	**Number of patients/animals**
1	Abdullah et al. (6)	Randomized controlled clinical trial	6 months	24 humans
2	Bhawal et al. (2)	Experimental study	1 and 2 weeks	24 rabbits
3	Costa et al. (3)	Experimental study	3 months	8 sheeps
4	Daher et al. (10)	Case report	4 years	1 human
5	Dallabrida et al. (12)	Experimental study	3 months	8 sheeps
6	Damlar et al. (9)	Experimental study	8 weeks	8 pigs
7	Joshi et al. (4)	Prospective randomized clinical, radiographic, and histological study	4 months	15 humans
8	Kim et al. (11)	Retrospective study	6–8 months	81 humans (103 maxillary sinuses)
9	Klein et al. (7)	Experimental study	2–6 weeks	84 mice
10	Knabe et al. (8)	Experimental study	2 weeks to 18 months	36 sheeps
11	Okada et al. (5)	Prospective observational study	6 months and 2 years	30 humans
12	Putri et al. (13)	Experimental study	4 months	3 rabbits

Of the twelve articles selected, seven were experimental studies, one was a retrospective study, one was a randomized controlled clinical trial, one was a randomized prospective clinical, radiological, and histological study, one was a prospective observational study, and one was a case report.

Of the twelve studies, six articles reported clinical, radiological, and histological evidence of bone neoformation using pure β - TCP phase, two showed radiological and histological analysis, one presented clinical and histological analysis, one revealed only histological study, one reported μ-CT, SEM (scanning electron microscopy) and histological analysis, and one reported only a SEM analysis. More information on the clinical, radiological, and histological findings can be found in Table 2.

**Table 2:** Articles in alphabetical order of authors included in the systematic review showing the methodology and biomaterials used, as well as the effectiveness of the studies demonstrated by clinical, radiological and/or histological evidence of new formed bone after placement of the implant.

**References**	**Methods/Biomaterials**	**Clinical/Radiological/Histological evidence of new bone formation/Residual implant**
Abdullah et al. (6)	Preservation of the socket after tooth extraction. Group A–defect filled with 1% melatonin + β - TCP Group B–defect filled with pure β - TCP phase	All had neoformed bone, residual biomaterial, and well-vascularized non-inflamed connective tissue. Group (A) showed the highest mean values of bone density, height, and bone width, followed by Group (B). The histological study reported more bone maturation in group A than in group B. Complete bone maturation occurred later in group B than in group A.
Bhawal et al. (2)	Implantation of biomaterial in defects created in the knee joints (lateral epicondyle of the femur). Group 1–6 animals without implant Group 2–6 animals with HFIP-F implant^a^ Group 3–6 animals with implant A-F^b^ Group 4–6 animals with β - TCP implant^c^	The A-F scaffold, as well as the β - TCP, have a more evident osseoinductive capacity than the HFIP-F scaffold. Osseointegration was observed between native tissue and new tissue within the bone defects. In histological sections, osteoclast-like cells were observed on the surface of β - TCP. New bone formation was observed from residual bone toward β - TCP more frequently after 2 weeks compared to the 1-week post-surgery group. Areas rich in connective tissue/bone marrow cells were observed between the bone substitute particles and the residual bones. However, there was new bone among the β - TCP granules.
Costa et al. (3)	Creation of 3 bone defects in each tibia of each animal and filled with: 2 proximal defects–HA^d^; HA/β - TCP 60/40^e^; 2 medial defects–autogenous bone graft only; 2 distal defects–β - TCP; HA/Al_2_O_3_ 5%^f^	HA, β - TCP and HA/β - TCP showed great osseoregenerative capacity. HA/β - TCP appears to be better for a long-term outcome. 5% HA/Al_2_O_3_ is not a good answer. The β - TCP showed intermediate results regarding the osseoregenerative capacity.
Daher et al. (10)	β - TCP only for maxillary sinus floor elevation.	They exhibited bone maturation, remodeling, and development similar to the Havesian bone morphology. Presence of multiple lamellar bone remodeling sites with a configuration similar to the Haversian cortical.
Dallabrida et al. (12)	Creation of 3 bone defects in each tibia of each animal. Filling: 2 with bone autograft (both proximal defects of each tibia); 1 with AH (right middle tibia); 1 with β - TCP (left middle tibia); 1 with 80/20 HA/β - TCP or 70/30 HA/β - TCP (distal right tibia); 1 with HA/β - TCP 20/80 or HA/β - TCP 30/70 (left distal tibia).	All synthetic bioceramics showed good capacity for osseoinduction, osseoconduction and osseointegration, promoting good ability to stimulate bone formation. In all materials, the graft granules and implants were well-integrated with varying degrees of active bone remodeling occurring within 3 months. All biomaterials showed varying degrees of absorption over the evaluation period, the most promising being the proportions of HA/β - TCP 20/80 and HA/β - TCP 30/70, followed by β - TCP, and then by the proportions of HA/β - TCP 80/20, HA/β - TCP 70/30 and HA, respectively.
Damlar et al. (9)	Five bone defects in the frontal bone of the skull of each animal: 3 defects tested with three types of β - TCP; 1 defect tested with allograft as a positive control; and 1 defect tested with blood clot as a negative control.	All groups showed new bone formation. Among the β - TCP groups, Poresorb M^®^ had better bone formation, through better % of osseoconductivity, TbTh and TbWi; while Cerasorb^®^ had the worst result for these parameters. Cerasorb^®^ had the highest TbSp; and Kasios^®^ had the worst % residual graft. Regarding the percentage of bone conduction, Poresorb^®^ presented the best values, while Cerasorb^®^ presented the worst.
Joshi et al. (4)	Preservation of the alveolar edges after 3 extracted teeth: 1 well filled with β - TCP 1 alveolus filled with ATG 1 alveolus not grafted	All alveoli had new bone formation. The ATG-grafted sites showed harder consistency than the β - TCP grafted sites and less reduction in the height and width of the vertical bone crest. Histologically, β - TCP showed less osteoid formation and poor integration with newly formed bone. The connective tissue around the β - TCP was poorly vascularized and there was infiltration of inflammatory cells.
Kim et al. (11)	Allografts were implanted in 40 sinuses, xenografts in 26 sinuses, and a mixture of allografts and xenografts in 35 sinuses. A mixture of allografts and alloplastics was implanted in 2 sinuses. The allografts used were lyophilized bone allograft, Orthoblast^g^, Tutoplast Spongiosa Microchip^h^, Allotis^i^ or Grafton^j^. The xenografts used were inorganic bovine bone matrix^k^, Biocera^l^ coated with calcium phosphate nanocrystal or OCS-B^m^. The alloplastic used was pure-phase β - TCP^n^.	Clinically 97.09% (100 of 103) of all maxillary sinuses showed complete bone healing at the bone windows. Three cases had infection (2.91%). Radiographically, all sinus walls (97.09%) were reconstructed. Histologically, all samples evaluated (97.09%) showed bone formation without formation and invagination of fibrous connective tissue.
Klein et al. (7)	Part 1–Alveolar bone regeneration model. Bone defects created. 1 filled with allograft, 1 filled with β - TCP alone, 1 left unfilled. Part 2–Orthodontic tooth movement in a local restoration model. Bone defects. 1 filled with β - TCP, 1 filled with allograft, only 1 filled without filling.	Bone volume and bone trabeculation were shown to be reduced in the β - TCP group compared to the allograft group and the non-grafted group at 2 and 4 weeks after graft use but were similar at 6 weeks. Graft particles could be detected 2 weeks after grafting for the β - TCP group, and 2 and 4 weeks for the allograft. The presence of a higher number of osteoclasts was observed in the β - TCP group at 2 and 4 weeks compared to allograft and control. OTM behaved similarly in the two grafted groups but was worse compared to the non-grafted control.
Knabe et al. (8)	4 critical defects were created in each animal's scapula: 1 filled with Si-CAOP^o^; 1 filled with Si-TCP^p^; 1 filled with β - TCP^q^; 1 defect left unfilled.	After 2 weeks and after 1 month, the defects grafted with Si-CAOP exhibited significantly greater bone area, bone-particle contact, osteogenic marker expression, and significantly smaller particle area than the defects grafted with Si-TCP and β - TCP. At 3 and 6 months, all materials showed excellent defect regeneration, with additional bone remodeling at 12 and 18 months.
Okada et al. (5)	Maxillary sinus floor elevation. Use of pure phase β - TCP in all patients.	Implant osseointegration achieved in all patients. Different degrees of newly formed bone replacing β - TCP in the second surgery. Radiographically, the mean bone volume and implant height decreased with time.
Putri et al. (13)	A total of 6 defects were made in 3 rabbits, being 1 distal femur defect of 6.1 mm on the medial condyle (bilaterally) of each animal. All filled with porous compressed or dense pure-phase β - TCP^r^ of 6 mm in diameter x 3 mm in height.	μ-CT images of the dense and porous β - TCP blocks, acquired 4 weeks after reconstruction show residual β - TCP in both dense and porous β - TCP blocks. The CT value was higher in the case of the dense β - TCP block. The histomorphometric results showed that a good amount (9.2 ± 3.1%) of the porous β - TCP was resorbed, and the amount of new bone was 18.9 ± 5.5% after 4 weeks; this value is much higher than that of the dense β - TCP block. In the case of the dense β - TCP block, resorption was only 0.2 ± 0.1%, and the amount of new bone formed was limited (0.1 ± 0.1%). In dense β - TCP block no cells or tissues are observed in the interior of the block. In contrast, osteoblasts, osteoclasts, osteocytes, red blood cells, bone tissue, and fibrous tissues are observed within the porous β - TCP block.

*^a^HFIP-based Silk Fibroin (HFIP-F)*;

*^b^Water-based silk fibroin scaffold (A-F)*;

*^c^β - TCP - (Taihei Chemical Industrial Co., Nara, Japan)*;

*^d^Hydroxyapatite*;

*^e^hydroxyapatite/β - TCP 60/40 composite*;

*^f^5% hydroxyapatite/alumina nanocomposite; Poresorb M^®^ (Curasan AG, Kleinostheim, Germany); Cerasorb^®^ (Lasak, Prague, Czech Republic); Kasios^®^ (Kasios, L'Union, France); TbTh, trabecular thickness; TbWi, trabecular width; TbSp, trabecular separation*;

*^g^Orthoblast, (IsoTis OrthoBiologics Inc., Irvine, CA)*;

*^h^Tutoplast Spongiosa Microchip, (Pures; Zimmer Dental, Carlsbad, CA)*;

*^i^Allotis, (Biotis, Seoul, Korea)*;

*^j^Grafton, (Osteotech, Eatontown, NJ)*;

*^k^Inorganic bovine bone matrix (Bio-Oss; Geistlich Pharma AG, Wolhusen, Switzerland)*;

*^l^Biocera, (Oscotec, Cheonan, Korea)*;

*^m^OCS-B, (Nibec, Seoul, Korea)*;

*^n^β, TCP - (Cerasorb M; Curasan, Kleinosthein, Germany); OTM, orthodontic tooth movement*.

*^o^Osseolive, glassy crystalline material with main crystalline phase Ca_2_KNa(PO_4_)_2_ with a mixture of 4% sodium and magnesium silicate*;

*^p^Ceracell, pure β - TCP phase with a 4% sodium and magnesium silicate mixture*;

*^q^Cerasorb M, pure phase of β - tricalcium phosphate = β - TCP [Ca_3_(PO_4_)_2_]*;

*^r^β - TCP, 100 (Taihei Chemical Industry Co., Japan); β - TCP, Beta - tricalcium phosphate; ATG, autogenous tooth graft*.

Regarding the countries where the experimental works were carried out, two were in Japan (2, 13), two in Brazil (3, 12), one in Turkey (9), one in Israel (7), and one in Germany (8). The case report was carried out in the USA (10). And finally, clinical studies took place in Egypt (6), India (4), South Korea (11), and Japan (5), respectively.

All twelve studies included in this systematic review reported a significant increase in the percentage of new bone formation when β - TCP in its pure-phase was used, including two randomized controlled trials. Some of the articles described this increase as a percentage of the volume density of the newly formed bone and one article also described it as a bone mineral content in milligrams. None of the articles reported complications, except (9, 11). Only six articles mentioned residual biomaterial at the implanted site (Table 3). When cost analysis was searched, of the eleven original studies that reported scientific evidence of new bone formation using pure-phase β - TCP, two mentioned costs, but the information was very vague (Table 3). They only mentioned the best cost-benefit using the β - TCP implant but did not say the exact value of the costs of implants or procedures related to their use.

**Table 3:** Effectiveness of β - TCP implant shown through newformed bone, volumetric density, bone mass content, and residual graft demonstrated in some articles.

**References**	**Effectiveness^**a**^%**	**BV/TV^**b**^**	**Residual implant**	**Cost**	**Complications**
Abdullah et al. (6)	100	NM	NM	NM	N
Bhawal et al. (2)	100	~20% in 2 weeks	NM	NM	N
Costa et al. (3)	100	NM (Show classification of ++++/0 to 4+)	NM	NM	N
Daher et al. (10)	100	NM	NM	NM	N
Dallabrida et al. (12)	100	NM	NM	NM	N
Damlar et al. (9)	100	Cerasorb^c^-41.28 ± 4.02%. Kasios^d^-43.85 ± 3.07%. Poresorb^e^-48.71 ± 2.56% All after 8 weeks.	*Cerasorb - 29.42 ± 6.29%; *Kasios - 38.85 ± 4.87%; *Poresorb - 37.14 ± 3.38%	NM	No postoperative complications, but 1 was excluded due to frontal sinus perforation during defect preparation.
Joshi et al. (4)	100	NM	NM. Only that a few particles were left.	Material with better cost-benefit but does not show numbers.	N
Kim et al. (11)	97.09	NM	NM	NM	Infection−2.91% (3 patients). Cracked bone in 2 other cases. 12 sinus perforations during osteotomies.
Klein et al. (7)	100	~60% in 4 and 6 weeks.	All absorbed in 4 weeks.	Reduced costs but does not show numbers.	NM
Knabe et al. (8)	100	0.75% in 2 weeks; ~43% in 1 month; ~62% in 3 months; ~68% in 6, 12, and 18 months.	~64% in 2 weeks. ~32% in 1 month. ~21% in 3 months. ~16% in 6 months. ~12% in 12 months. ~8% in 18 months.	NM	N
Okada et al. (5)	100	NM	24.4% in 6 months and 45.1% in 2.5 years.	NM	The implant tip protruded into the maxillary sinus by ~70% of cases (41/58 implants) in 2 years.
Putri et al. (13)	100	Porous β - TCP block - 18.9 ± 5.5% in 4 months. Dense β - TCP block - 0.1 ± 0.1% in 4 months.	99.8 ± 0.1% in dense β - TCP in 4 months. 90.8 ± 3.1% in porous β - TCP in 4 months.	NM	NM

*Implant costs, complications, and other intercurrences were also mentioned when present*.

*^a^Effectiveness through new bone formation*;

*^b^Volumetric density (VB/TV) in percentage; VB, bone volume (VB); TV, tissue volume*.

^*^p <0.0001 (statistically significant);

*^c^Cerasorb^®^ (Lasak, Prague, Czech Republic)*;

*^d^Kasios^®^ (L'Union, France); and*

*^e^Poresorb M^®^ (Curasan AG, Kleinostheim, Germany). NM, not mentioned; N, none*.

For this reason, we found another review article, which is not included in this systematic review, but mentions the price-brand relationship in its data, and for this reason we included here the prices of three brands of β - TCP compared in this article for the knowledge of the reader only. The estimated cost of pure phase β - TCP granules were US $ 63.00–US $ 73.60. With the articles selected for this systematic review, we were not able to corroborate the prices of all pure-phase beta tricalcium phosphate brands, but only the value of two of them (Table 4). There were no mentions of additional indirect costs associated with using this technology.

**Table 4:** Price ratio of β - TCP implants with brand names and granule sizes.

**Chemical composition**	**Commercial name**	**Size^**#**^**	**Price U.S. $**	**References**
β-TCP	Cerasorb^a^	0.5	73.60	**Bluesky Bio** http://blueskybio.com/store/pure-phase-b-tcp
β-TCP	Premier TCP^b*^	0.5	70.00	**Osseous Technologies of America** http://www.osseoustech.com/promotions/2012-ao/
β-TCP	Synthograft^c^	0.5	63.00	**eBiologics dental** http://www.ebiologicsdental.com/SearchResults.asp?searching=Y&scrt=13&search=synthograft&show=1&page=6

*References about their prices and links to companies that market them are also provided*.

*^a^Cerasorb (Lasak, Prague, Czech Republic)*;

*^b^Premier Biomaterials (Riverside Business Park, Nenagh, Co. Tipperar, Ireland)*;

*^c^SynthoGraft (Arborway, Boston, MA, USA)*;

*^#^In grams or cubic centimeters*;

^*^Implant was not used in any of the articles selected in the systematic review. Javaid and Esfandiari, 2013.

Other relevant attributes related to β - TCP implantation were also described. Of the twelve articles included in this review, six emphasized the use of β - TCP as a form of granules. Only one article used it as powder and only one used as a block shape. Four of them did not mention the shape of the implant, but there is a trade name associated with it where readers can search for. Only six of the twelve papers showed particle sizes and only six were explicit about the porosity of the granules. The size of the bone defects created for implant insertion was explained only in nine of the twelve studies. The mentioned data can be seen in Table 5.

**Table 5:** Brand, shape, porosity, and particle size of the implants used by the authors.

**References**	**Name of implant (Brand)**	**Shape of implant**	**Particle size**	**Particle porosity**	**Critical size of bone defect**
Abdullah et al. (6)	IngeniOs β - TCP^a^	Granules	NM	NM	22.03 ± 2.96 mm (alveolus depth) x 8.50 ± 2.60 mm (oral-lingual width)
Bhawal et al. (2)	β - TCP-100^b^	Powder, ρ = 3.14 g/cm3	20–200 μm	NM	3.25 mm (diameter) x 4.95 mm (depth)
Costa et al. (3)	NM	Granules	NM	46.07–54.44%	6 mm (diameter)
Daher et al. (10)	SynthoGraft^c^	Granules	50–500 μm	NM	Not mentioned size in mm (osteotomy in the position of the right maxillary first molar)
Dallabrida et al. (12)	Tricalcium -phosphate - β^d^	Granules	200 - 500 μm	7.57 ± 0.82%	6 mm (diameter)
Damlar et al. (9)	Cerasorb M^e^ Kasios^f^ Poresorb^g^	All 3 are granules	Cerasorb M: micro-pores smaller than 50 μm, and macro-pores from 50 to 500 μm. Kasios: micro-pores of 1–5 μm and macro-pores of 200–500 μm. Poresorb: 1–5 μm micro-pores and 100 μm macro-pores.	Cerasorb M - 62% Kasios - 60–80% Poresorb - 30–40%	1 cm (diameter) x 4 mm (depth)
Joshi et al. (4)	SyboGraft^h^	NM	NM	NM	Does not mention size in mm (implants were inserted in alveolar sockets after tooth extractions in the maxilla and mandible)
Kim et al. (11)	Cerasorb M	NM	NM	NM	Vertical anterior osteotomy - 2–3 mm distal to the vertical anterior wall of the maxillary sinus. Distal osteotomy - 15 mm away from the vertical anterior osteotomy. Vertical osteotomy height was ~10 mm
Klein et al. (7)	β - TCP. But it doesn't mention the brand used.	NM	NM	NM	Tooth socket widened with a reamer and drill, resulting in a 4-sided defect of ~15 μl
Knabe et al. (8)	Cerasorb M	Granules	1,000-2,000 μm	65% (pores size 0.1–500 μm)	8 mm (diameter) and 8 mm (depth)
Okada et al. (5)	OSferion^i^	NM	NM	NM	NM (only that it was an elevation of the maxillary sinus floor in the premolar and molar region)
Putri et al. (13)	β - TCP - 100^j^	Powder (3.07 g/cm^3^) compressed into granules and later into final blocks	Porous β - TCP block: connected macropores of 40–160 μm. Micropores not mentioned. Dense β - TCP block: No presence of macropores, but includes micropores. Do not mention the particle size.	Porous β - TCP block (58.1 ± 1.7%) and pore volume of 0.32 cm^2^/gr. Dense β - TCP block (10.9 ± 2.3%) and pore volume of 0.01 cm^3^/gr.	6.1 mm (diameter)

*The sizes of bone defects created for implantation of β - TCP are also shown in this table*.

*^a^Bioactive Synthetic Bone Particles, Zimmer Biomet Dental, USA*;

*^b^Taihei Chemical Industrial Co., Nara, Japan*;

*^c^Bicon, LLC; Boston, MA*;

*^d^Department of Mechanical Engineering, CCT, UDESC/Joinville/SC Brazil*;

*^e^Curasan AG, Kleinostheim, Germany*;

*^f^Kasios, L'Union, France*;

*^g^Lasak, Prague, Czech Republic*;

*^h^T Plug, Eucare Pharmaceuticals Unip. Ltd., Chennai, Tamil Nadu, India*;

*^i^Olympus Terumo Biomaterials Corp., Japan*;

*^j^β - TCP - 100 (Taihei Chemical Industry Co., Japan); NM, not mentioned*.

## Discussion

The systematic review of articles using β - TCP in its pure-phase to assess osseointegration between the years 2011 and 2021 generated few articles (12 articles) after databases searching. In the initial phase of the search, more than 14.500 articles were found in the databases with the selected keywords, but in the end, after the inclusion and exclusion criteria, only 12 articles met the prerequisites for selection. However, we expected a greater number of articles, a fact that may be related to the more frequent use of β - TCP associated with some other type of material, forming composites. Many of these articles were excluded from this systematic review because they did not fit the chosen search terms.

The association of biomaterials could even be noticed in some articles of this review, which despite presenting results with the use of pure β - TCP as a control, also brought us results from other types of biomaterials not mentioned here, or even comparisons between them. However, those who used pure-phase ceramic were only included in this review due to the keywords selected and relation to β - TCP and the results mentioned about it, which could be used independently in the context of the article (2–4, 6, 8, 11, 12).

It was found that the pure-phase use of this ceramic is currently more used in dentistry (5–7, 10, 11), especially in cases related to bone augmentation of the floor of the maxillary sinuses for later placement of titanium implants and for alveolar filling, as described by some authors in randomized and prospective clinical studies with human patients (4–6).

Most of the articles included in this systematic review were classified as experimental studies (7 articles). This fact shows us that even if some of the researchers carry out studies using new types and associations of biomaterials to promote bone growth and bone differentiation throughout the implants micropores, there are still authors doing research using pure-phase β - TCP ceramic to assess osseoconduction and osseointegration. This may show that despite this ceramic has been already extensively studied, maybe there is still a lack of information and characteristics about it that could be learnt from continuing research about this topic (2, 3, 7–9, 12, 13).

This could be verified by the different species that were used in the experimental models listed above, such as sheep, rabbits, pigs, and mice, as well as the observational study periods, which ranged from 1 week to 18 months after the implantation of β - TCP. In addition, we could also verify the various types of bones used in the experiments for implantation of bioceramics, such as the bone of the maxilla, tibia, femur, scapula, and frontal bone of the skull. An interesting fact is that in all these experimental animal studies using bone defects for implantation of β - TCP, the bone defects created for implantation were only cylindrical holes, with diameters varying up to 6 mm in a single cortical bone and performed using a drill. It was not identified in any of the articles the use of β - TCP, the occurrence of osseoconduction and bone neoformation, which were performed in bone defects with diameters >6 mm and compromising the 2 cortical bones, or even critical segmental defects in long bones that would create a total loss of contact between the ends of the ostectomized bone.

As for the other 5 articles in which the patients were human, all were included in clinical studies (4–6, 11), except for one of them, which was a clinical case report (10), and in all these cases the articles were related to alveolar filling or maxillary sinus floor elevation in dentistry. It is important to mention that no article selected for this search period showed the use of β - TCP in long bones, spine, or other bones, nor was performed in the areas of orthopedics or traumatology, either clinically or experimentally, as in cases of vertebral fusions, filling of bone cysts, bone replacement in cases of neoplasms, bone non-unions, or even for cases of fractures with great bone loss that required an implant for bone replacement and concomitant preservation of bone length.

Despite the good results obtained with the use of pure-phase β - TCP in relation to bone neoformation by the osseoconduction process, in which all authors reported 100% osseointegration with the use of this bioceramic, some of the articles showed that the association of other types of materials with osteogenic characteristics and/or osseoinductive properties to β - TCP, or even other forms of composites, showed superior results compared to the use of pure phase β - TCP, either by a greater local bone production or even for a quicker time in the osseointegration process through cell growth at the implant interface (2–4, 6, 8, 11, 12).

In all twelve studies, clinical, radiographic, or histological signs of bone neoformation were evidenced when β - TCP was used in its pure-phase, and only two presented complications, being (2.91%) (11) and (11.1%) (9), respectively, but not due to the use of the implant itself, but due to problems associated with the surgical technique.

Regardless of the design of the studies included in this systematic review, the effectiveness of using this ceramic has been proven. It was observed that the bone tissue invades the environment in which the ceramic granules are implanted, and adheres to them due to their porous characteristics, which are replaced over time by new bone, which proves its bioactive and osseoconductive activity.

The β - TCP implant also did not cause immunological reactions or inflammatory response when in contact with the host tissue, indicating its biocompatible properties (7).

In the histological evaluations, what was described were different degrees of bone maturation depending on the implant used, and different degrees of bone maturation with time, as described by Abdullah et al. (6). And despite the formation of new bone, there was still an amount of residual implant that remained after a long period of implantation (4, 6, 13). The first author shows that there was formation of well-vascularized connective tissue and no presence of inflammatory cells at the site, differing from the study conducted by Joshi et al. (4), which showed that despite the formation of new bone around the β - TCP implant, there was less osteoid formation in relation to the other study group. In addition, the connective tissue around the β - TCP implant was poorly vascularized and presented several inflammatory cells, also differing from the other author. The third author, comparing dense and porous β - TCP blocks, observed no cells or tissues in the interior of the implant. In contrast, within the porous block, osteoblasts, osteoclasts, osteocytes, red blood cells, bone tissue, and fibrous tissues were observed for this shape of implant. The presence of osteoblasts, osteoclasts, and osteocytes within the implant shows that there is an active bone remodeling process in the interior of the block. Furthermore, the presence of red blood cells indicates the formation of capillary vessels and/or Harvesian canal-like structures. And the presence of bone and fibrous tissues indicates the usefulness of interconnected structure as a path for tissue penetration (13).

The osseoconduction process takes place through the constant formation of osteoblasts that form around the implant and osteoclasts that destroy the implant over time, opening space for the formation of new bone (9, 13). This process was also described by Bhawal et al. (2), Klein et al. (7), Putri et al. (13), who showed in histological sections the presence of osteoclastic cells on the surface of the β - TCP implant, as well as the formation of Havers' canals during the bone healing phase. In another study, it was demonstrated that bone maturation, bone remodeling, and bone formation occurred with characteristics similar to those of the Haversian system in multiple sites where β - TCP was implanted (10).

Throughout the process of bone formation and remodeling, it was observed that bone/implant integration occurs over time, being a dynamic process that occurs as new bone forms at the same time as the implant is degraded and reabsorbed (12). It is known that the faster the biomaterial is absorbed, the more bone is deposited between the implant gaps and faster is the bone maturation process at the site. The same author (12) also showed that there is a different degree of absorption for each implant or even for combined proportions of implants over time. He had demonstrated that the pure phase β - TCP presented a better degree of absorption when compared to the β - TCP associated with HA in the proportions 20/80 and 30/70, respectively. On the other hand, when compared with the proportions 80/20 and 70/30, respectively, for each material, the pure phase β - TCP presented worse absorption rate and degree of osseoconduction and osseointegration in relation to the composite.

Other recent study showed that there is a clear difference in β - TCP resorption depending on block porosity (13). The denser the block, the lower is its resorption. Only 0.2 ± 0.1% was the absorption in dense β - TCP blocks, and in contrast, 9.2 ± 3.1% was the absorption in porous blocks (13).

In porous blocks, the β - TCP exposure to body fluids is dissolved and supplies Ca^2+^ PO${}_{4}^{3-}$ to the adjacent tissues. This β - TCP dissolution together with calcium and phosphate ions are all enhanced by the acid conditions provided by osteoclasts. This dissolution is higher in porous blocks than in dense blocks undoubtedly, because they have larger specific surface area. In addition, Ca^2+^ and PO${}_{4}^{3-}$ concentrations will induce osteogenesis (13).

Another particularly interesting factor observed in this systematic review is that in all the articles included in the study, the ceramic format used by the authors was always that of granules with different granulometries and porosities, except in the article of (2), in which the β - TCP used was in powder and article (13), in which β - TCP was used as dense or porous blocks. No other papers used β - TCP in a different format such as paste, wedge, or compact cylinders to fill in defects and promote osseointegration.

It has been reported that granulation can interfere with the osseoconduction process (2, 9, 13). Larger particles would allow for earlier bone regeneration, forming a more stable structure compared to materials that have smaller particles. And materials with smaller micropores have better osteoconductivity. These properties make them more effective in forming new bone (9, 13).

There is one recent study (13) that used a block shaped β - TCP in distal femoral condyle that reported excellent results for implant resorption and new bone formation after 4 weeks of implantation, and the authors considered them as good indicators for its potential use as an artificial bone substitute. But we must take into consideration that this was an experimental study and the authors used only 3 rabbits, which may not provide valid results because the low number of animals used as a model. Between all experimental studies mentioned in this systematic review, this was the one with the lowest number of experimental animals used.

One question we raise in this regard is whether the use of β - TCP cylinders or compact blocks could be used in bone replacement in cases of critical segmental bone defects in long bones, such as extensive fractures with great loss of bone tissue, for example. Cases in which there would be the need for a more compact material to restore the bone defect longitudinally, while maintaining the original length of the bone, and biomechanically being more rigid to withstand forces over time. In this way, what we would see was the union of the two bone segments, proximal and distal, at the interfaces with the ends of the implant. Thus, we imagined that osseoconduction would occur from the proximal and distal ends of the implant and over the surface of the implant, toward the center of the implant. This could open perspectives for further studies, initially using the pure-phase β - TCP, but also, later associating the implant in question with other types of materials, forming composites for analysis of osseointegration in critical segmental defects in veterinary medicine.

However, as observed in the PRISMA flowchart, during the inclusion/exclusion process of the selected articles, when the keyword “long bones” was inserted, there was a considerable decrease in the number of articles, but none of them fit the eligibility criteria after reading the titles, abstracts, and objectives. This factor may indicate that there is a perspective of action in this area using β - TCP to correct large bone losses in fractures, excision of bone tumors and bone non-unions in veterinary medicine.

Comparing different brands of pure phase β - TCP, it was found that even though all of them presented new bone formation, there was a difference in the values presented in relation to osteoconductivity. This was verified by Damlar et al. (9), when the author compared 3 different types of pure phase β - TCP, Poresorb M^®^, Kasios^®^ and Cerasorb^®^, and showed that the first one presented the best results for bone formation, presenting a higher percentage of osseoconductivity and better values of bone thickness (TbTh) and bone width (TbWi), while Cerasorb^®^ presented the worst indexes for the same evaluated parameters. And regarding the rate of implant resorption over time, Kasios^®^ was the worst among the three.

All the studies that cited the effectiveness of this biomaterial through radiological evaluation showed good bone integration and consolidation, with different values of bone volume and trabeculation, especially for those clinical studies in humans in which maxillary sinus floor elevation and filling of dental sockets were performed. However, an important information given by one of the authors (5) was in relation to the mean bone volume and the thickness (height) of the maxillary sinus floor after 2 years of β - TCP implantation in 30 patients. The author found that in several of these patients (70%), radiographically there was a decrease in the evaluated parameters mentioned above when compared to the radiographic study of the 6-month postoperative period, which may be related to bone resorption that occurred over time. This means that, due to this bone resorption, there was exposure of the tip of the titanium implants inserted in the maxillary bone associated with β - TCP, but without complications. This study, unlike most of the others mentioned, is one of those that evaluated for the longest time patients who received β - TCP ceramic implants to increase bone volume. Therefore, we cannot know whether in the other selected articles, specifically referring to experimental studies in which the observation time was <6 months, whether such a process of bone volume reduction would occur after osseointegration. These findings may be associated with the natural process of bone remodeling that occurs in a physiological way.

On the other hand, another study, a case report of 1 patient who was evaluated for 4 years after a maxillary sinus floor augmentation procedure using β - TCP, did not evidence such bone absorption due to time (5). Despite presenting the results with a much longer time than in the previous article, we must take into account that the report presents only one patient. Perhaps if there were more patients in this study group, the results could be different.

We did not find in the evaluated articles the prices related to the β - TCP implants used. As different brands were used, a comparison between them would be necessary to stipulate the cost-benefit. As none presented numbers, but only a vague mention of “lower costs,” we turned to another article that was initially excluded from the initial research, because during its reading it was the only one in which we found a price list with some of the implant brands used.

We believe that we had some limitations in our systematic review, most of them related to the heterogeneity of the studies. Different animal species, as well as the bones used as experimental models, including long bones, calvaria bone and maxillofacial bones were used in combination with various implant application techniques in different species, as well as the size of the bone defects created. Regarding the β - TCP implant, there is heterogeneity regarding its sizes, porosity, brands, amount of biomaterial implanted in the defect sites. Regarding the chosen method of observation time, there is heterogeneity in terms of follow-up periods. Times ranged from 1 week to 4 years.

In addition, in relation to the osseointegration process and bone healing, which are the main objectives of this systematic review, they were approached in different ways by the studies, with some evaluated by radiographic images, others by histological analysis, others by clinical evaluation, and others evaluated through combinations of these different methods.

Even knowing that all of them reported positive changes in the implanted area with good results in bone formation and integration, this limits our study when we try to make a more adequate comparison between the parameters evaluated, which may cause a bias in the study.

Due to the lack of quantitative evidence in some of the studies, as well as the heterogeneity observed regarding the brands of biomaterials, granule sizes, different locations, and sizes in the realization of the bone defect for implant placement, patient species, follow-up periods and lack of information of some comparable items, it was not possible to perform a meta-analysis on the eleven articles included in this systematic review.

## Conclusion

β - TCP in its pure-phase is a biomaterial that has been tested and used for cases of bone augmentation and replacement for decades. In general, after autogenous grafts, we can say that β - TCP is the most reliable and widely used synthetic biomaterial category of materials for grafting in dentistry and maxillofacial surgery. But there is lack or no information about its use as biomaterial for filling critical segmental defects of long bones in veterinary medicine. In this area, only experimental studies in small defects were carried out, with no clinical cases performed in animals with a longer observation time. β - TCP can produce predictable, sustainable, and adequate bone formation, with minimal infection rates and low patient morbidity, essential characteristics to search for when considering bone filling and bone replacement. More future clinical studies showing the exact and standardized values of measurements that evidence bone healing, as well as studies showing other formats (such as cylinders and blocks), and clinical uses of β - TCP alone or in association with other biomaterials are needed to evaluate their osseoinductive, osseoconductive and osseointegrative properties, as well as to determine possible new comparisons and quantitative analyzes for further evaluations on the subject.

## Data Availability Statement

The original contributions presented in the study are included in the article/supplementary material, further inquiries can be directed to the corresponding authors.

## Author Contributions

DG and MS conceived and designed this systematic review and coordinated the research and selected the articles. DG and LM performed the searches in the databases, read and selected the articles for inclusion/exclusion of the same and the relevant data of each article, and wrote the article. All authors critically reviewed the manuscript and approved the final version.

## Funding

Funding was provided by grant 04/2022, Paraíba State Research Foundation (FAPESQ).

## Conflict of Interest

The authors declare that the research was conducted in the absence of any commercial or financial relationships that could be construed as a potential conflict of interest.

## Publisher's Note

All claims expressed in this article are solely those of the authors and do not necessarily represent those of their affiliated organizations, or those of the publisher, the editors and the reviewers. Any product that may be evaluated in this article, or claim that may be made by its manufacturer, is not guaranteed or endorsed by the publisher.
